# An Account of the Tabasheer

**Published:** 1791

**Authors:** Patrick Russell


XV. *An Account of the Tabaſheer. In a Letter from* Patrick Ruſſell, *M. D. F. R. S. to Sir* Joſeph Banks, *Bart. P. R. S.*—Vide *Philoſophical Tranſactions of the Royal Society of London. Vol.* LXXX. *for the Year* 1790. *Part.* II. 4to. London, 1791.

THE drug, which is the ſubject of the paper before us, has long been a medicine of high repute in the Eaſt, and is mentioned by all the Arabian Medical writers, as an important article of their materia medica.

To the Arabs and Turks it is known under the name of Tabaſheer only; and under that name alſo it is mentioned in the writings of the Arabian phyſicians.

In the Gentoo language it is called *Vedroo-paloo*, Bamboo milk; in the Malabar, *Mungel Upoo*, ſalt of Bamboo; and in the Warriar, *Vedroo Carpooram*, Bamboo Camphor.

Our author obferves, that Garcias ab Horto long ago pointed out a dangerous error, common to the old tranflators of the Arabian writers, refpecting this drug; Tabafheer, in the Latin verfions of Rhazis and Avicenna, being conftantly rendered *fpodium*, and this interpretation having been adopted by moft of the fubfequent tranflators of other Arabian medical writers.

The late Mr. Channing, when engaged in the tranflation of Rhazis on the fmall-pox, applied, it feems, to our author, then in Syria, for fuch information as he might be able to collect on the fubject of Tabafheer at Aleppo. Dr. Ruffell, accordingly tranfmitted to him various fpecimens of the drug, together with feveral extracts relative to it, from books found in the Aleppo libraries; but he is now convinced, he tells us, that much of the drug commonly vended in Turkey is fictitious or adulterated.

The Arabian medical writers generally agree in fuppofing the Tabafheer to be a production of the Indian reed; more efpecially of fuch as have fuffered from fire, kindled by the friction of the reeds one againft the other, an accident that is faid to happen frequently in the dry feafon.

Our

Our author has been aſſured, he tells us, by ſeveral mountaineers, with whom he has converſed on the ſubject, that the bamboo is not the only tree ſubject to accidental ignition by friction; but theſe people added that they never looked for Tabaſheer in the half-burnt fragments of the bamboo, though they doubted not it might ſometimes be found there as well as in others.

Dr. Ruſſell is convinced that the genuine Tabaſheer is a production of the *Arundo Bambos* LINN. the *Ily* of the Hortus Malabaricus, and the *Arundo Indica arborea maxima, cortice ſpinoſo*, of Herman; but he thinks it no leſs certain that fire is not a neceſſary agent in its production.

He obſerves that the bamboo, in which the Tabaſheer is found, is vulgarly called the Female Bamboo, and is diſtinguiſhed by the largeneſs of its cavity from the male. They are even ſaid, it ſeems, to be ſeparate trees; but this fact he has not been able to aſcertain.

Of the ſeven pieces of bamboo which accompanied the paper before us, four, we are told, were from the mountains near Vellore, and three from a ſpot diſtant about twenty miles from Vizagapatam, the place from which the author dates * his letter. The former were perfectly

* Nov. 26, 1788.

green green on their arrival at Madras; and the others were ſtill ſo when they came to his hands. Theſe, he obſerves, were all ſelected on a conjecture of their containing Tabaſheer, from a certain rattling perceived upon ſhaking the bamboo, as if ſmall ſtones were contained in the cavity. This, by the natives, is conſidered as an indication of Tabaſheer being contained in one or more joints of the bamboo, and, it ſeems, they are ſeldom diſappointed; but it does not always follow that there is no Tabaſheer where a rattling is not perceptible; for upon ſplitting a number of reeds, it was ſometimes remarked, that where the quantity of the drug was inconſiderable, it was found adhering ſo cloſely to the ſides of the cavity, as to prevent any rattling from being perceived upon ſhaking. In general, however, our author has found the rule of the natives, for chooſing the bamboo, a good one.

On examining one of the bamboos, conſiſting of ſix joints, from Vellore, Dr. Ruſſell found Tabaſheer only in two of the joints, and in theſe the quantity was various, amounting in the whole to about twenty-ſeven grains.

The quality alſo, he obſerves, was various. The particles reckoned of the firſt quality, and which which did not exceed four grains in weight, were of a bluiſh white colour, reſembling ſmall fragments of ſhells. They were harder than the others; and when rubbed between the fingers eaſily crumbled into a gritty powder, which had a ſlight ſaline teſtaceous taſte. The reſt were of a cineritious colour, rough on the ſurface, and more friable; and intermixed with theſe were ſome larger, light, ſpongy, particles, ſomewhat reſembling pumice ſtones. Dr. Ruſſell thinks it probable, that the Arabs, from theſe appearances of the drug, were led into the opinion already mentioned of its production.

The two middle joints, he obſerves, were of a pure white colour within, and lined with a thin film, and it was in theſe chiefly the Tabaſheer was found. The others, particularly the two upper joints, were diſcoloured within, and in ſome parts of the cavity was found a blackiſh ſubſtance in grains, or in powder, adhering to the ſides, the film being there obliterated.

Thirty-ſeven green bamboos, brought from the hills fifty miles diſtant from Vizagapatam, being carefully ſplit and examined, the reſult we are told, was as follows.

In nine out of the thirty-ſeven there were no veſtiges of Tabaſheer. In twenty-eight ſome were found in one, two, or three joints of each; but never in more than three joints of the fame bamboo. The quantity varied, but in all was inconfiderable; and the empty joints were fometimes contiguous, fometimes interrupted, indifferently.

Dr. Ruffell obferves that the whiter, fmooth, harder particles, when not loofe together with the others in the cavity, were moftly found adhering to the feptum that divides the joints, and to the fides contiguous; but never to the fides about the middle of the joints; and that inftead of being chiefly found at the lower extremity of the joint, as might be expected from the juice fettling there, they were found adherent indifferently to either extremity, and fometimes to both. In this fituation they formed a fmooth lining, which ufually was cracked in feveral places, and might eafily be detached with a blunt knife.

In fome joints, the Tabafheer was found thus collected at one or both extremities only, and in fuch no rattling was perceived upon fhaking the bamboo; but in general, we are told, while fome adhered to the extremities of the joint, other detached pieces were intermixed with the coarfer loofe particles in the cavity.

The

The quantity found in each bamboo is ſaid to have been very inconſiderable; the produce of the whole twenty-eight reeds, from five to ſeven feet long, not having much exceeded two drachms. The author therefore thinks it very probable that the Tabaſheer, though not abſolutely confined to certain regions, may be produced in greater abundance in ſome ſoils than in others; but that in all regions where the bamboo grows favourably, ſome proportion of it will be found, however it may vary in quality or quantity.

Rumphius, who very candidly acknowledges that he himſelf had not had opportunities of making enquiries on this ſubject, refers his readers to Garcias, who has remarked that the Tabaſheer is not found in all bamboos, nor in all the branches indiſcriminately; but only in thoſe growing about Biſnagur, Batecala, and one part of the Malabar coaſt.

With reſpect to Biſnagur our author has been informed in a letter from one of his medical friends at Hydrabad, "That though Tabaſheer be in great requeſt at Hydrabad, and "bears a high price, it is never brought "thither from Biſnagur; that ſome of what is "found in the Bazars is brought from the Ar-

" cour paſs in Canoul, and ſome from Emna-

" bad, at the diſtance of about eighty miles to

" the N. W.; but that the greateſt part comes

" from Maſulipatam.

" That there are two ſorts ſold in the Bazars;

" one at the rate of a rupee a drachm; the

" other, of inferior quality, at half the price;

" but that this is ſaid to be chiefly compoſed of

" burnt teeth and bones.

" That he was informed by a Perſee, who

" had been in Bengal, that the Tabaſheer was

" produced in great quantities at Sylhet, where

" it ſold by the pound from one rupee to one

" and a half, and formed a conſiderable article

" of trade from Bengal to Perſia and Arabia."

Dr. Ruſſell has procured ſome of the prime ſort of Tabaſheer from Hydrabad, and found it to differ materially from his own ſpecimens, not only in its ſuperior whiteneſs, and in its being leſs mixed with impure particles, but alſo in being much harder and heavier, and ſcarcely in any degree friable to the finger.

With reſpect to the ſuppoſed formation of Tabaſheer from the juice of the recent bamboo Dr. Ruſſell firſt quotes Rumphius, who remarks that in Amboina " Juniores arundines plerum-

" que in inferioribus ſuis nodis ſemi-repletæ

" utcunque sunt lympida aqua potabili, quæ,

" hisce in terris sensim evanescit, in aliis vero

" regionibus exsiccatur in substantian albam

" et calceam quæ *Tabaxir* vocatur."

Garcias, as our author observes, gives an account somewhat different from this. " Inter

" singula internodia liquor quidam dulcis ge-

" neratur, crassus veluti amylum congestum,

" et simili candore, interdum multus, nonnun-

" quam vero perpaucus. Sed non omnes arun-

" dines sive rami eum humorem continent. . . .

" . . . . Hic autem liquor concretus, interdum

" nigricans et cinereus invenitur, sed non ideo

" improbatur. Nam aut ob nimiam humidi-

" tatem, aut quod diutius ligno inclusus

" permanserit, hunc sibi colorem conciliat:

" non autem ob arborum incendium, veluti

" nonnulli putarunt. Siquidem in multis

" ramis, quos non contigit ignis, niger etiam

" invenitur *.

The existence of this fluid in the bamboo is known by shaking the joint. In a considerable number of bamboos Dr. Russell never found water in more than two joints, and generally not more than two or three drachms in each. The

* Cap. 12.

 largest largeſt quantity procured by him at one time was one ounce and a half; very few joints, in proportion, being found to contain any.

The fluid, he obſerves, was always tranſparent, but varied in conſiſtence; when thicker it had a whiter colour than uſual; when more dilute it differed little to the eye from common water, except that ſometimes it had a pale greeniſh caſt. Applied to the tongue it had a ſlight ſaline, ſub-aſtringent taſte, more or leſs perceptible in proportion to the conſiſtence of the fluid. After evaporation in the ſun, the reſiduum had a pretty ſtrong ſaline taſte, with leſs aſtringency. Some of the fluid, of a darkiſh colour, thickened in the reed to the conſiſtence of honey; while ſome, in another part of the reed, was perfectly white and almoſt dry; both, we are told, had the ſharp ſalt taſte, which the Tabaſheer itſelf loſes in a great degree by keeping.

From an ounce of fluid of a greeniſh caſt, and ſlight ſaline taſte, procured from green bamboos, our author obtained by ſlow evaporation ſmall particles of a whitiſh colour reſembling the inferior ſort of Tabaſheer.

As a further proof that this ſubſtance is formed in the cavity of the bamboo, we learn, from from our author's obſervations, that recent green bamboos, which, upon ſhaking, appeared to contain water in their cavity, loſt this appearance after ſtanding a few days; and that when ſplit, after they had ceaſed to give any ſound by ſhaking, ſometimes no fluid was found in the cavity. The interior thin pellicle, however, was diſcoloured, as if by recent moiſture; and in general ſome of the fluid, in a mucilaginous ſtate, remained at the lower part of the joint.

In the latter end of October, a green bamboo of five joints was brought to him, which appeared to contain both water and Tabaſheer. After three days, the ſound of water, upon ſhaking the reed, could hardly be perceived; and on the fifth day it was intirely imperceptible. Upon ſplitting the bamboo about half a drachm of the fluid, now thickened into a mucilage, was found at the bottom of the upper joint. The ſecond joint contained ſome perfect Tabaſheer looſe in the cavity. The third joint was empty, excepting a few particles of Tabaſheer which adhered to the ſides near the bottom. The fourth joint, at the bottom, contained above a drachm of a browniſh pulpy ſubſtance adherent. The laſt joint, in like manner, contained half a drachm of a fubftance thicker and harder in confiftence, and nearly of the colour of white wax.

This fpecimen, as the author obferves, exhibited at one view the progrefs of the Tabafheer through its feveral ftages.

In a poftfcript to this letter, dated Weymouth Street, July 16th, 1790, we are informed that four of the green reeds prefented to the fociety on the night the preceding account was read, having been carefully fplit, the contents, upon comparing them with the fpecimens fent from India, then on the table, were found to agree in all refpects, as well as with the defcription of the more recent ones given in this paper. *

XVI.

* In addition to the above account of the Tabafheer, from the Philofophical Tranfactions, the following extract of a letter, on the fame fubject, addreffed to the Editor of this work, will it is prefumed not be unacceptable to the reader; and more efpecially as it ferves to confirm the opinion of Dr. Ruffell with refpect to the formation of this fubftance. It is written by Mr. J. L. WILLIAMS, an ingenious Surgeon in the fervice of the Hon. Eaft India Company, in Bengal, and is dated at Benares, October 23, 1790.

'I have lately procured from the hills in this neighbourhood, 'a drug, fpecimens of which I fhall fend, by the fhips of this 'feafon, for your infpection.

